# Retraction: “Recovering with nature”: A review of ecotherapy and implications for the COVID-19 pandemic

**DOI:** 10.3389/fpubh.2022.1124835

**Published:** 2023-01-04

**Authors:** Frontiers Editorial Office

Following publication, the publisher has discovered that the corresponding author submitted false information that compromised the peer-review process. As the scientific integrity of the article cannot be guaranteed, and adhering to the recommendations of the Committee on Publication Ethics (COPE), the publisher therefore retracts the article.

The authors do not agree to this retraction.

This retraction was approved by the Chief Editors of Frontiers in Public Health and the Chief Executive Editor of Frontiers.

